# Reduced risk of chronic GVHD by low-dose rATG in adult matched sibling donor peripheral blood stem cell transplantation for hematologic malignancies

**DOI:** 10.1007/s00277-019-03884-8

**Published:** 2019-12-11

**Authors:** Liping Dou, Cheng Hou, Chao Ma, Fei Li, Xiaoning Gao, Wenrong Huang, Shuhong Wang, Chunji Gao, Li Yu, Daihong Liu

**Affiliations:** Department of Hematology, Chinese PLA General Hospital, Fuxing Road 28th, Haidian District, Beijing, 100853 China

**Keywords:** ATG, Stem cell transplantation, Peripheral blood, Graft-versus-host disease, Relapse

## Abstract

The optimal rabbit anti-thymocyte globulin (rATG) graft-versus-host disease (GVHD) prophylaxis regimen in matched sibling donor peripheral blood stem cell transplantation (MSD-PBSCT) remains to be elucidated. In this prospective study, we used low-dose rATG for GVHD prophylaxis in patients or donors aged ≥ 40 years with hematological malignancies receiving MSD-PBSCT. rATG was administered to 40 patients at an intravenous dose of 5 mg/kg divided over day 5 and day 4 before graft infusion. No graft failure occurred. Median times to leukocyte engraftment and platelet engraftment were 11.0 days and 13.9 days. The cumulative incidence of grades 2–4 and grades 3–4 acute GVHD at day +100 was 30.0% and 2.6%. The 2-year cumulative incidence of extensive chronic GVHD and severe chronic GVHD was 11.4% and 14.7%. 93.5% (29/31) of patients had discontinued immunosuppressive medication within 3 years after transplantation. The 2-year cumulative incidence of transplant-related mortality (TRM) and relapse was 14.0% and 22.6%. The cumulative incidence of cytomegalovirus reactivation, Epstein–Barr virus reactivation, and fungal infection was 22.3%, 12.9%, and 12.5%. Kaplan–Meier estimates for overall survival, disease-free survival, and GVHD-free and relapse-free survival 3 years after transplantation were 68.9%, 68.9%, and 54.0%. rATG for GVHD prophylaxis is tolerable and efficacious at a 5 mg/kg total dose administered over 2 days (days −5 to −4) in patients receiving allogeneic MSD-PBSCT.

## Introduction

Chronic graft-versus-host disease (GVHD) increases the risk for long-term morbidity, poor quality of life, and death after matched sibling donor (MSD) stem cell transplantation [1]. Risk factors for chronic GVHD (cGVHD) include receipt of a peripheral blood stem cell graft and a history of acute GVHD (aGVHD) [2]. There is an urgent need to reduce the incidence of severe cGVHD and improve the quality of life in patients who have undergone MSD peripheral blood stem cell transplantation (MSD-PBSCT) [3]. We have reported that rabbit anti-thymocyte globulin (rATG) (Sanofi, Paris, France) may effectively and safely reduce the incidence and severity of cGVHD in patients receiving myeloablative conditioning before peripheral blood stem cell transplantation from haploidentical donors [4]. The 3-year cumulative incidence of extensive and severe cGVHD was 41.5% and 21.2% in patients receiving MSD-PBSCT (without rATG) compared with 17.1% (*P* = 0.017) and 5.8% (*P* = 0.049) in patients receiving stem cells from haploidentical donors [4]. Our ATG GVHD prophylaxis regimen in unmanipulated haploidentical PBSCT includes rATG given at a dose of 10 mg/kg over 4 days (days −5 to −2) before graft infusion. The incidence of Epstein–Barr virus (EBV) and cytomegalovirus (CMV) antigenemia and EBV-associated post-transplantation proliferative disease (PTLD) was higher in patients receiving stem cells from haploidentical donors compared with that of patients receiving MSD-PBSCT. Donor age ≥ 40 years vs. younger donors was associated with a higher incidence of aGVHD grades 2–4 (*P* = 0.02) and treatment-related mortality (TRM) (*P* = 0.022) [4].

The use of rATG is a promising strategy for GVHD prophylaxis [5–11]. However, engraftment, immune reconstitution, and GVHD prevention must be carefully balanced. The optimal ATG regimen for GVHD prophylaxis in MSD-PBSCT remains to be elucidated [8]. Due to a high incidence of cGVHD in patients or donors aged ≥ 40 years, rATG was prospectively added to the preparative regimen in MSD-PBSCT. In this prospective study (ClinicalTrials.gov Identifier: NCT02677181), we investigated the feasibility of using low-dose rATG (Sanofi, Paris, France: preparation containing polyclonal immunoglobulins obtained from hyper-immune sera of rabbits immunized with human thymocytes) for GVHD prophylaxis in patients or donors aged ≥ 40 years with hematological malignancies receiving MSD-PBSCT, compared with no use of rATG in patients or donors aged 14 to 40 years. rATG was administered at an intravenous dose of 5 mg/kg divided over day 5 and day 4 before graft infusion, which is half of the dose used by our group to reduce the risk of severe aGVHD in patients receiving hematopoietic stem cell transplantation without ex vivo T cell depletion from haploidentical donors [8].

## Methods

This study was conducted in accordance with the principles of the Declaration of Helsinki and approved by the Ethics Committee of the Chinese People’s Liberation Army (PLA) General Hospital. All patients provided written informed consent.

### Study design

Consecutive patients with myelodysplastic syndrome (MDS)/acute leukemia (AML/ALL) who received allogeneic MSD-PBSCT with low-dose rATG (Sanofi, Paris, France) in combination with cyclosporine, mycophenolate, and short-term methotrexate for GVHD prophylaxis at our center between June 1, 2013, and August 1, 2019, were eligible for this study. First-line GVHD prophylaxis for HLA-identical sibling transplant patients or donors aged 40 to 65 years was low-dose rATG (Sanofi, Paris, France) in combination with cyclosporine, mycophenolate, and short-term methotrexate. First-line GVHD prophylaxis for HLA-identical sibling transplant patients or donors aged 14 to 40 years was cyclosporine, mycophenolate, and short-term methotrexate without rATG. All patients received myeloablative conditioning regimens. The conditioning regimen for MSD-PBSCT consisted of busulfan (Otsuka Pharmaceutical Company, China; 3.2 mg/kg/day intravenously, days −10 to −8), carmustine (Jinyao Tianjin Pharmaceutical Company, China; 250 mg/m^2^ intravenously, day −5), cytarabine (Pfizer Pharmaceutical Company, USA; 2 g/m^2^/day intravenously, days −7 to −6), and cyclophosphamide (Baxter Pharmaceutical Company, USA; 60 mg/kg/day intravenously, days −4 to −3).

Inclusion criteria were (1) patients or donors aged 14 to 65 years; (2) diagnosed with acute leukemia or myelodysplastic syndrome; and (3) indicated for MSD-PBSCT.

The number of CD34-positive cells and lymphocyte subpopulations in the graft was analyzed. Multi-parameter flow cytometry was performed [9]. Median lymphocyte counts stratified into CD3^+^, CD4^+^, CD8^+^, and CD56/CD16^+^ subpopulations were determined (Wilcoxon rank sum test).

### HLA matching and graft selection

Patients and donors were typed by means of high-definition PCR Luminex and sequence-based typing methods at HLA-A, HLA-B, HLA-C, DRB1, and DQB1 [10]. All pairs were matched at these loci.

### rATG and GVHD prophylaxis

Mobilized peripheral blood stem cells were transplanted [11, 12].

Patients in the ATG group were treated with rATG 1.5 mg/kg for 4–6 h on day −5 and rATG 3.5 mg/kg for 4–6 h on day −4 for a total dose of rATG 5 mg/kg prior to transplant.

Patients in both groups received cyclosporine 3 mg/kg intravenously from day −10 until oral refeeding, targeting minimum concentration levels of 150 to 200 ng/mL for the first month. After 6 months, the dose of cyclosporine was tapered by 25% every 2 weeks in all patients except for those who had post-transplant relapse, in which case, the dose of cyclosporine was tapered after relapse. Tacrolimus was used in cases of cyclosporine intolerance or toxicity. Methotrexate15 mg/m^2^ was administered on day +1, and methotrexate 10 mg/m^2^ was administered on days +3, +6, and +11. Mycophenolate mofetil 500 mg was administered orally twice daily from days −10 to +30.

Antimicrobial and antiviral prophylaxis was routinely administered. Blood samples were tested for EBV and CMV using polymerase chain reaction (PCR) testing [13, 14].

### GVHD therapy

All aGVHD patients received a total daily dosage in two divided doses of methylprednisolone 2 mg/kg/day for 7 consecutive days, followed by a gradual reduction in dose, and a taper over 8 weeks [15, 16].

### Immune monitoring

Immune reconstitution after transplantation was studied in a subset of patients who consented to have peripheral blood samples collected on days +30, +60, +90, +180, +240, and +360. Multi-parameter flow cytometry was performed [9]. Median lymphocyte counts stratified into CD3^+^, CD4^+^, CD8^+^, and CD56/CD16^+^ subpopulations were determined at each time point (Wilcoxon rank sum test) and analyzed with time-dependent multivariable Cox proportional hazards regression models.

### Definitions and end points

All surviving patients were followed-up from the date of transplantation to August 1, 2019. Days before graft infusion were documented as “−,” and days after last stem cell infusion were documented as “+.”

Primary endpoints were incidence of cGVHD, TRM, and relapse. Secondary endpoints were engraftment, incidence of aGVHD, overall survival (OS), disease-free survival (DFS), and GVHD-free and relapse-free survival (GRFS). Only patients with successful engraftment after transplantation were included in the aGVHD analysis. Patients who survived at least 100 days after transplantation were included in the cGVHD analysis [9, 15, 16]. The date of neutrophil recovery after transplantation was defined as the first of 3 consecutive days with an absolute neutrophil count > 0.5 × 10^9^/L [17]. The date of platelet recovery after transplantation was defined as the first of 7 consecutive days with an absolute platelet count > 20 × 10^9^/L without the aid of transfusion.

Patients were assigned a disease risk using the Disease Risk Index as previously described [18]. Diagnosis and grading of aGVHD and cGVHD were performed according to standard criteria [16]. cGVHD was classified as “limited” or “extensive” according to the Seattle criteria [15, 16]. cGVHD was classified as “mild” or “moderate” or “severe” according to the National Institutes of Health (NIH) criteria [15, 16]. OS was defined as the time from transplantation to death from any cause. DFS was defined as survival with no evidence of relapse or disease progression. Relapse was defined as the presence of peripheral blood blast or > 5% bone marrow blasts and/or reappearance of underlying disease. GRFS was defined as the time onset of grade 3 to 4 aGVHD, moderate to severe cGVHD, or relapse/disease progression/death. TRM was defined as death without evidence of relapse or disease progression, with relapse as a competing event. Quality of life in survivors was measured with the Karnofsky Performance Status Scale.

### Statistical analysis

Statistical analysis was performed using R statistical software (cmprsk package) and SPSS 20.0 Treatment group comparisons were performed using chi-square tests for categorical data and Mann–Whitney *U* tests for continuous variables. Cumulative incidence was estimated for TRM, relapse, and GVHD (grades 2 to 4 or 3 to 4 aGVHD and cGVHD of any severity or extensive). The probability of developing aGVHD or cGVHD was depicted by determining the cumulative incidence with aGVHD or cGVHD without relapse as competing risks. Gray’s test was used to assess the difference between treatments. The 95% CI for the differences was calculated using the Wilson score method. OS, DFS, and GRFS were computed with the Kaplan–Meier method. Univariate and multivariate analyses were performed with Cox proportional hazards regression analysis.

Prognostic factors were diagnosis, patient age at transplantation, donor-recipient sex matching, status at time of transplantation (complete remission versus other), time from diagnosis to transplantation (< 6 months vs. ≥ 6 months), nucleated and CD34^+^ cells dose/kg, and blood group and compatibility. Predictors with *P* values < 0.2 on univariate analyses were included in the multivariate analysis. *P* < 0.05 was considered significant.

## Results

### Patient characteristics

A total of 72 consecutive patients were enrolled in this study (Table 1). Of these, 2 patients were excluded because of progressive disease (1 patient) or the donor withdrew (1 patient). The final analysis included 70 patients (40 patients in the ATG group and 30 patients in the no-ATG group). At the time of transplantation, the patients with AML/ALL were in complete remission (CR1) following conventional therapy or salvage therapy, while the patients with MDS-EB-2 were untreated. Median follow-up was 996 days (range, 271–1825 days). Patients and donors in the ATG group were older than those in the no-ATG group (patients: ATG group 46.9 years vs. no-ATG group 29.7 years; donors: ATG group 48.8 years vs. no-ATG group 29.8 years). No difference was observed in other baseline clinical characteristics between the two groups, including the percentage of high-risk disease according to the Disease Risk Index [18].

**Table 1:** Clinical features of SCT recipients and donors

Characteristic	ATG group	No ATG group	*P* value
No. of patients	40	30	
Patient’s age, median, years (range)	46.9 (40–62)	29.7 (12–39)	0.001
Gender			0.334
Male	22	13	
Female	18	17	
Time between diagnosis and stem-cell transplantation—days			0.055
Median (range)	172.9 (50–344)	313 (59–2014)	
Diagnosis—no. (%)			0.431
Acute myeloid leukemia	17	18	
Acute lymphoid leukemia	14	6	
MDS	9	6	
Disease status at transplantation			0.323
Untreated MDS-AML	9	5	
CR1	31	25	
High cytogenetic risk—no. (%)	18 (45.0%)	10 (33.3%)	0.725
Disease Risk Index—no./total no. (%)			0.268
Low	4 (10.0%)	2 (6.7%)	
Intermediate	26 (65.0%)	15 (50.0%)	
High	10 (25.0%)	13 (43.3%)	
Very high	0 (0.0%)	0 (0.0%)	
Conditioning regimen			0.685
Modified Bu/Cy	38	29	
Modified Bu/Flu	2	1	
Donor’s age, median, years (range)	48.8 (40–61)	29.8 (11–39)	0.001
Donor-recipient ABO match			0.454
Match	18	21	
Major mismatch	6	4	
Minor mismatch	4	5	
Bidirectional mismatch	3	0	
Donor-recipient gender match			0.859
Female to male	14	12	
Female to female	10	5	
Male to female	10	7	
Male to male	8	6	
Graft			
MNCs, median, × 10^8^/kg (range)	9.2 (6.2–14.6)	8.0 (5.1–12.6)	0.063
CD34^+^, median, × 10^6^/kg (range)	3.6 (1.6–8.2)	3.6 (2.3–9.3)	0.547
B cell, median, × 10^6^/kg (range)	21.1 (14.3–34.6)	19.9 (7.9–29.4)	0.669
T cell, median, × 10^6^/kg (range)	84.6 (11.8–265.4)	93.3 (46.5–248.7)	0.710
CD4^+^T cell, median, × 10^6^/kg (range)	46.1 (0.3–171.5)	49.3 (1.3–133.8)	0.818
CD8^+^T cell, median, × 10^6^/kg (range)	33.1 (4.2–72.7)	36.9 (1.8–115.3)	0.692
NK cell, median, × 10^6^/kg (range)	14.8 (0.2–97.6)	9.2 (0.2–25.9)	0.576

*ATG*, anti-T lymphoglobulin; *CR*, complete remission; *MDS-AML*, AML evolution from MDS; *NHL*, non-Hodgkin lymphoma; *NR*, non-remission; *SCT*, hematopoietic stem cell transplantation; *WBC*, white blood cell; *MNC*, mononuclear cells; *NK cell*, natural killer cell

### Graft failure and engraftment

Patients in the ATG group received G-CSF mobilized PBSC with MNCs 9.2 (6.2–14.6) × 10^8^/kg and CD34^+^ cells 3.6 (1.6–8.2) × 10^6^/kg. Patients in the no-ATG group received MNCs 8.0 (5.1–12.6) × 10^8^/kg and CD34^+^ cells 3.6 (2.3–9.3) × 10^6^/kg. There were no differences in the counts of the lymphocyte subpopulations in the grafts between the two groups (Table 1). No graft failure was observed. One patient (3.2%) in the ATG group died of severe infection on day +14 before engraftment. One patient (3.3%) in the no-ATG group developed prolonged isolated thrombocytopenia and died of aGVHD on day +71 without platelet engraftment. Sustained myeloid engraftment with full donor chimerism was achieved in all tested patients. Median times to leukocyte engraftment and platelet engraftment in the ATG group were 11.0 days (range, 8 to 16 days) and 13.9 days (range, 9 to 30 days), respectively (Table 2). Median times to leukocyte engraftment and platelet engraftment in the no-ATG group were 11.0 days (range, 9 to 15 days) and 15.8 days (range, 9 to 22 days), respectively (Table 2).

**Table 2:** Rates of engraftment, infection, acute and chronic GVHD, and other complications after allogeneic peripheral blood stem cell transplantation from HLA-identical sibling

Variable	ATG group (*n* = 40)	No ATG group (*n* = 30)	*P* value
Graft failure—no. (%)	0	0	
Prolonged isolated thrombocytopenia—no. (%)	0	0	
Days to engraftment—median (range)			
Absolute neutrophil count ≥ 0.5 × 10 9/L	11.0 (8–16)	11.0 (9–15)	0.517
Platelet count ≥ 20 × 10 9/L	13.9 (9–30)	15.8 (9–22)	0.710
Infectious complication—no. (%)	15 (37.5)	12 (40.0)	0.018
Cytomegalovirus reactivation—no. (%)	11 (27.5)	3 (10.0)	0.070
Epstein–Barr virus reactivation—no. (%)	5 (12.5)	1 (3.3)	0.039
Post-transplantation lymphoproliferative disorder—no. (%)	0	0	
Acute GVHD within 100 days after transplantation—no. (%)	15 (37.5)	15 (50.0)	0.786
Overall grades of acute GVHD—no. (%)			0.453
0	25 (62.5)	15 (50.0)	
1	2 (5.0)	2 (6.7)	
2	12 (30.0)	10 (33.3)	
3	1 (2.5)	3 (10.0)	
4	0 (0)	0 (0)	
2–4	13 (32.5)	13 (43.3)	
3–4	1 (2.5)	3 (10.0)	
Chronic GVHD			0.153
Day of onset			
Median (range)	524 (150–957)	380 (112–889)	
Severity according to revised Seattle criteria—no. (%)			0.033
Limited	7 (17.9)	6 (21.4)	
Extensive	6 (15.4)	9 (32.1)	
Severity according to NIH criteria—no. (%)			0.085
Mild	7 (17.9)	4 (14.3)	
Moderate	5 (12.8)	6 (21.4)	
Severe	1 (2.6)	5 (17.9)	

*ATG*, anti-T lymphoglobulin; *CR*, complete remission; *MDS-AML*, AML evolution from MDS; *NHL*, non-Hodgkin lymphoma; *NR*, non-remission; *SCT*, hematopoietic stem cell transplantation; *WBC*, white blood cell; *MNC*, mononuclear cells

Chronic, limited, and extensive GVHD are defined according to the Seattle criteria

Chronic, mild, moderate, and severe GVHD are defined according the NHI criteria

### Acute and chronic GVHD

**Fig. 1:**
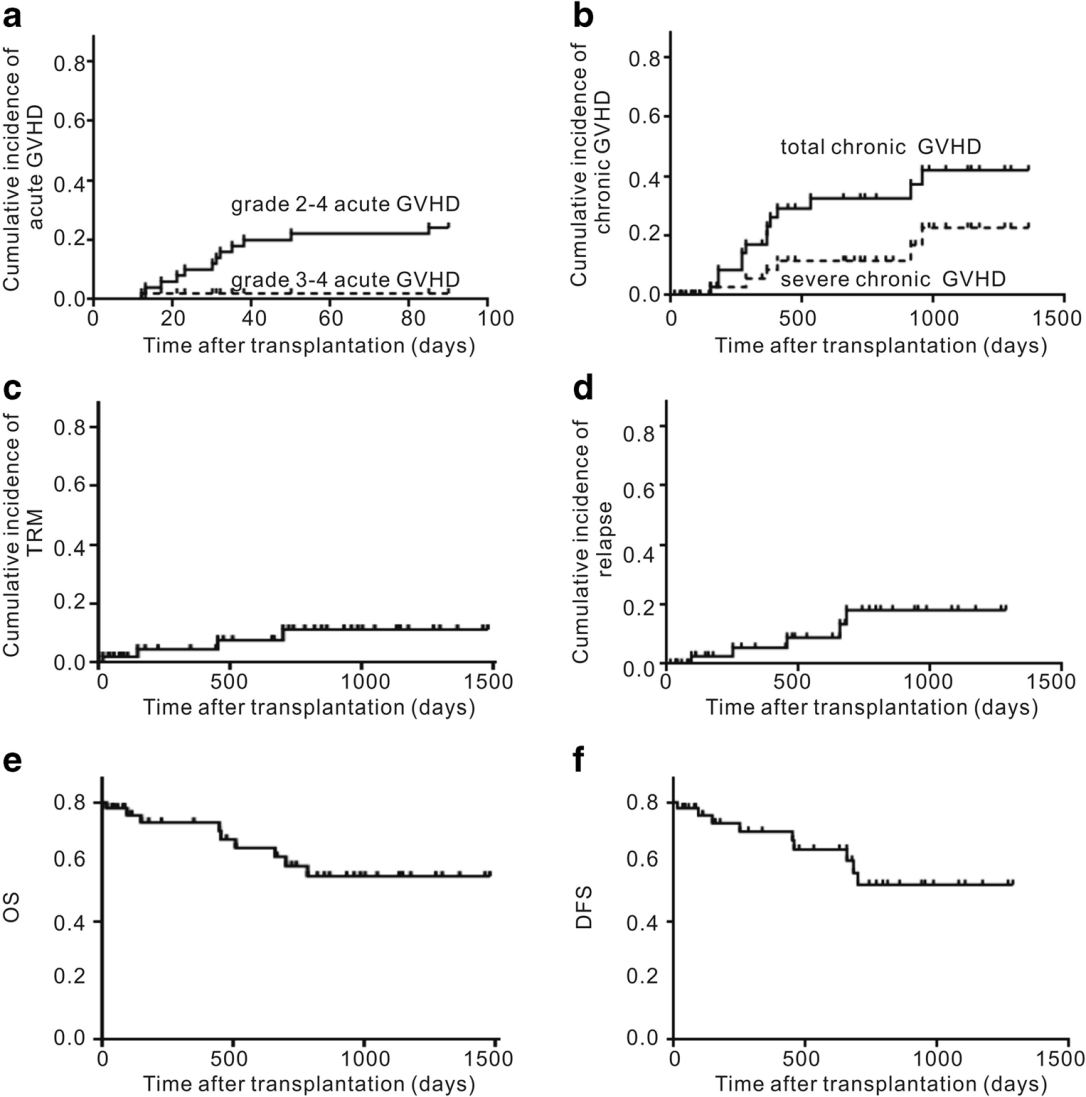
Cumulative incidence of aGVHD, cGVHD, TRM, relapse, OS, and DFS after MSD-PBSCT with low-dose rATG in combination with cyclosporine, mycophenolate, and short-term methotrexate for GVHD prophylaxis. GVHD, graft-versus-host disease; TRM, treatment-related mortality; OS, overall survival; DFS, disease-free survival

In the ATG group, 39 patients were engrafted and survived. Among these, 15 (38.5%) patients developed grades 1–4 aGVHD (grade 1, *n* = 2; grade 2, *n* = 12; grade 3, *n* = 1) at a median 30 (range, 13–85) days after transplantation. The cumulative incidence of grades 2–4 and grades 3–4 aGVHD was 30.0% (95% CI, 18.0–50.5%) and 2.6% (95% CI, 2.5–18.6%) at day +100, respectively (Fig. 1a). On univariate analysis, no risk factors were associated with the occurrence of > grade 2 aGVHD (Table 2). Two patients with aGVHD died. Causes of death were disease relapse (*n* = 1, grade 2 aGVHD) and pneumonia (*n* = 1, grade 3 aGVHD).

**Fig. 2:**
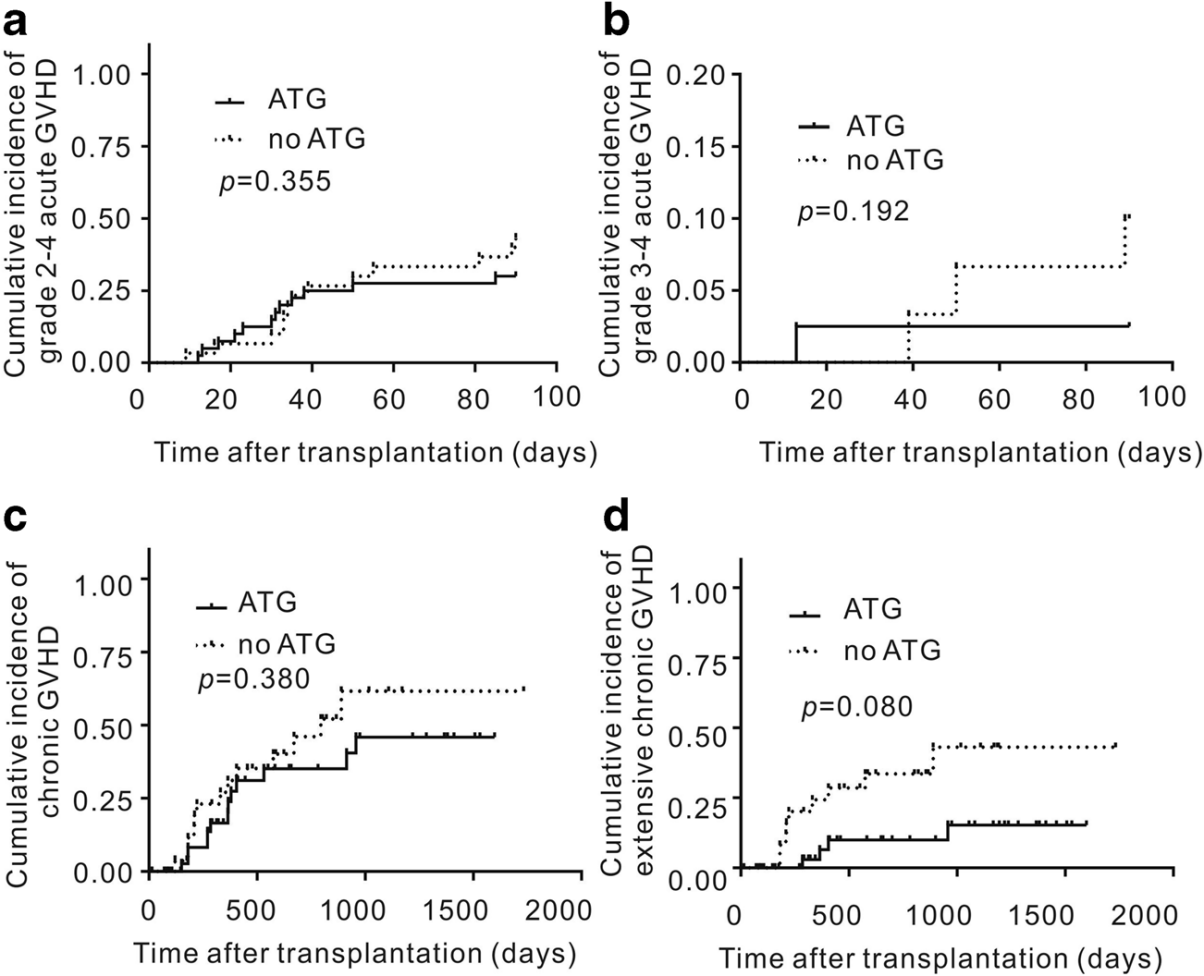
Cumulative incidence of aGVHD and cGVHD after MSD-PBSCT with low-dose rATG or no rATG in combination with cyclosporine, mycophenolate, and short-term methotrexate for GVHD prophylaxis. GVHD, graft-versus-host disease

There was no difference in the incidence of aGVHD among the 69 patients who were engrafted and survived (39 patients in the ATG group vs. 30 patients in the no-ATG group). The cumulative incidence of grade 2–4 aGVHD was 30.0% (95% CI, 18.0–50.5%) in the ATG group and 40.0% (95% CI, 22.4–57.0%) in the no-ATG group at day +100 (*P* = 0.355; Fig. 2a). The cumulative incidence of grade 3–4 aGVHD was 2.6% (95% CI, 2.5–18.6%) in the ATG group and 10.0% (95% CI, 2.5–23.9%) in the no-ATG group at day +100 (*P* = 0.192; Fig. 2b). In the no-ATG group, 7 patients with aGVHD died. Causes of death were disease relapse (*n* = 4), thrombotic microangiopathy (*n* = 1), and pneumonia (*n* = 2).

In the ATG group, a total of 13 (32.5%) patients developed cGVHD, with a median time to onset of +524 (range, +150–957) days. Five of the 13 patients with cGVHD had preceding aGVHD. The 1-year, 2-year, and 3-year cumulative incidences of cGVHD were 25.1% (95% CI, 19.6–26.8%), 40.5% (95% CI, 21.3–61.0%), and 52.4% (95% CI, 31.0–70.0%), respectively, and the 1-year, 2-year, and 3-year cumulative incidences of severe cGVHD were 7.6% (95% CI, 7.0–14.9%), 14.7% (95% CI, 14.5–21.6%), and 22.4% (95% CI, 21.0–37.0%), respectively (Fig. 1b). The 1-year, 2-year, and 3-year cumulative incidences of extensive cGVHD were 7.6% (95% CI, 7.0–14.9%), 11.4% (95% CI, 11.2–28.6%), and 18.8% (95% CI, 17.9–36.9%), respectively (data not shown). On univariate analysis, no risk factors were associated with the occurrence of cGVHD (Table 2). On multivariate analysis, significant predictors of cGVHD were female donor and male recipient (hazard ratio, 60.43; 95% CI, 2.72 to 134.62; *P* = 0.009) and a low infused CD34^+^ cell dose (hazard ratio, 0.54; 95% CI, 0.31 to 0.92; *P* = 0.02) (Table 4). Two patients with cGVHD died. Causes of death were relapse after cGVHD responded to treatment (*n* = 1) and pneumonia (*n* = 1). Eleven patients with cGVHD were alive at the last follow-up. Within 3 years after transplantation, 93.5% (29/31) of patients had discontinued immunosuppressive medication.

cGVHD was absent in 42 (62.7%) patients (27 (69.2%) patients in the ATG group vs. 15 (53.5%) patient in the no-ATG group), clinically limited in 13 patients (19.4%; 7 (17.9%) patients in the ATG group vs. 6 (21.4%) patients in the no-ATG group), and clinically extensive in 15 patients (22.4%; 6 (15.4%) patients in the ATG group vs. 9 (32.1%) patients in the no-ATG group) (Table 2). The 3-year cumulative incidence of cGVHD was 37.3% (95% CI, 17.8–57.0%) in the ATG group and 52.3% (95% CI, 28.3–71.7%) in the no-ATG group (Fig. 2c; *P* = 0.380). The overall 3-year cumulative incidence of extensive cGVHD alone was 19.5% (95% CI, 5.4–40.1) in the ATG group and 33.6% (95% CI, 15.4–53.0) in the no-ATG group (*P* = 0.080; Fig. 2d).

cGVHD severity according to the NIH criteria was mild in 11 patients (16.4%; 7 (17.9%) patients in the ATG group vs. 4 (14.3%) patients in the no-ATG group), moderate in 11 patients (16.4%; 5 (12.8%) patients in the ATG group vs. 6 (21.4%) patients in the no-ATG group), and severe in 6 patients (8.9%; 1 (2.6%) patient in the ATG group vs. 5 (17.9%) patients in the no-ATG group). Among these 28 patients, 13 patients with cGVHD had prior aGVHD (5 patients in the ATG group vs. 8 patients in the no-ATG group). On univariate and multivariate analyses, no-ATG was a significant risk factor for extensive cGVHD (univariate analysis: hazard ratio, 3.3; 95% CI, 1.3–10.0; *P* = 0.024; Table 3; multivariate analysis: hazard ratio, 13.0; 95% CI, 1.3–128.2; *P* = 0.028; Table 4). Eight patients (3 patients in the ATG group vs. 5 patients in the no-ATG group) with cGVHD died due to relapse after cGVHD responded to treatment (*n* = 2) or pneumonia (*n* = 6). Twenty patients with cGVHD were alive at the last follow-up.

**Table 3:** Univariate analysis of cumulative incidence for the risk factors of transplant outcomes in all patients in the ATG group

	Grades II–IV aGVHD		Grades III–IV aGVHD		Chronic GVHD		Sever chronic GVHD		TRM		Relapse		OS		DFS	
	% (95% CI)	*P*	% (95% CI)	*P*	% (95% CI)	*P*	% (95% CI)	*P*	% (95% CI)	*P*	% (95% CI)	*P*	% (95% CI)	*P*	% (95% CI)	*P*
Disease Risk Index		0.66		0.76		0.31		0.79		0.23		0.23		0.04		0.05
Low	25.0 (0.0–57.4)		0.0 (0.0–0.0)		66.7 (0.0–93.3)		0.0 (0.0–0.0)		5.0(0.1–15.8)		0.0(0.0–0.0)		50.0 (12.5–100.0)		50.0 (12.5–100.0)	
Intermediate	34.6 (13.5–50.6)		3.9 (0.0–10.9)		56.2 (24.6–74.5)		19.3 (0.0–37.5)		5.6 (0.3–23.3)		10.7 (1.6–28.8)		83.7 (68.2–100.0)		83.1 (67.3–100.0)	
High	20.0 (0.0–41.3)		0.0 (0.0–0.0)		50.0 (0.0–87.5)		50.0 (0.0–87.5)		22.9 (2.7–54.8)		38.6 (6.8–71.3)		34.3 (12.0–97.6)		32.1 (10.6–97.1)	
High cytogenetic risk		0.99		0.51		0.22		0.81		0.29		0.83		0.79		0.67
Low	33.7 (0.0–77.1)		0.0 (0.0–0.0)		100.0 (100.0–100.0)		0.0 (0.0–0.0)		5.3 (0.1–10.7)		0.0 (0.0–0.0)		100.0 (100.0–100.0)		100.0 (100.0–100.0)	
Intermediate	30.0 (6.7–47.5)		0.0 (0.0–0.0)		51.3 (14.2–72.4)		16.7 (0.0–35.9)		5.0 (0.3–21.0)		19.3 (4.3–42.4)		75.6 (57.2–100.0)		73.9 (54.5–100.0)	
High	29.4 (4.1–48.1)		5.9 (0.0–16.4)		45.4 (0.0–72.1)		31.8 (0.0–62.4)		16.7 (2.3–42.6)		17.7 (2.4–44.9)		64.3 (41.2–100.0)		58.3 (32.9–100.0)	
Remission status		0.89		0.08		0.73		0.47		0.04		0.85		0.1		0.16
Complete remission	30.0 (11.5–44.6)		0.0 (0.0–0.0)		47.2 (18.5–65.8)		9.7 (0.0–21.8)		5.7 (0.3–23.7)		14.5 (3.4–33.7)		78.8 (62.1–99.9)		77.8 (60.6–100.0)	
Advanced stage of disease	30.0 (0.0–53.4)		10.0 (0.0–26.8)		62.5 (0.0–86.9)		40.0 (0.0–70.7)		30.0 (6.3–59.3)		20.0 (2.4–49.8)		50.0 (26.9–92.9)		50.0 (26.9–92.9)	
Time from diagnosis to transplant		0.07		0.44		0.48		0.27		0.59		0.83		0.53		0.34
> 6 months	6.7 (0.0–18.5)		0.0 (0.0–0.0)		69.7 (0.0–93.1)		54.6 (0.0–88.8)		17.4 (2.1–45.2)		18.5 (2.3–47.1)		64.2 (40.7–100.0)		48.5 (19.9–100.0)	
< 6 months	44.0 (20.7–60.4)		4.0 (0.0–11.4)		48.7 (16.9–68.3)		14.1 (0.0–30.9)		10.9 (1.7–29.9)		16.9 (3.9–37.9)		72.0 (53.9–96.3)		71.5 (53.1–96.3)	

*P value

*ATG*, anti-thymocyte globulin; *95% CI*, cumulative incidence 95% CI; *DFS*, disease-free survival; *GVHD*, graft-versus-host disease; *OS*, overall survival

**Table 4:** Multivariate analysis of OS or DFS for the risk factors of transplant outcomes in all patients in the ATG group

Event	HR	95% CI	*P*
OS			
High Disease Risk Index			
No	1		
Yes	5.19	1.38–19.47	0.01
DFS			
High Disease Risk Index			
No	1		
Yes	5.29	1.41–19.88	0.01
Chronic GVHD			
Female donor and male recipient			
No	1		
Yes	60.43	2.72 to 134.62	0.01
Number of CD34 cells in the graft			
Low (less than × 106/kg)	1		
High (more than × 106/kg)	0.54	0.31–0.92	0.02

*P value

*ATG*, anti-thymocyte globulin; *95% CI*, cumulative incidence 95% CI; *DFS*, disease-free survival; *GVHD*, graft-versus-host disease; *OS*, overall survival

In the ATG group, among the 39 patients who were engrafted and survived, 3 patients (7.7%) developed late grade 2 aGVHD. Of these, one patient had persistent (liver) late aGVHD, one patient had recurrent late aGVHD (lower gastrointestinal tract, day +103), and one patient had de-novo late aGVHD (skin, day +310). No patients in the ATG group with late aGVHD died. One patient in the no-ATG group had recurrent late aGVHD (lower gastrointestinal tract, day +125).

### Toxicity, TRM, and relapse

In the ATG group, among the 40 patients who received allogeneic MSD-PBSCT, 15 (37.5%) patients experienced infectious complications, including CMV reactivation (27.5% [11/40] patients), Epstein–Barr virus reactivation (20.0% [8/40] patients), and fungal infection (12.5% [5/40] patients). No post-transplantation lympho-proliferative disorder was recorded (Table 2).

**Fig. 3:**
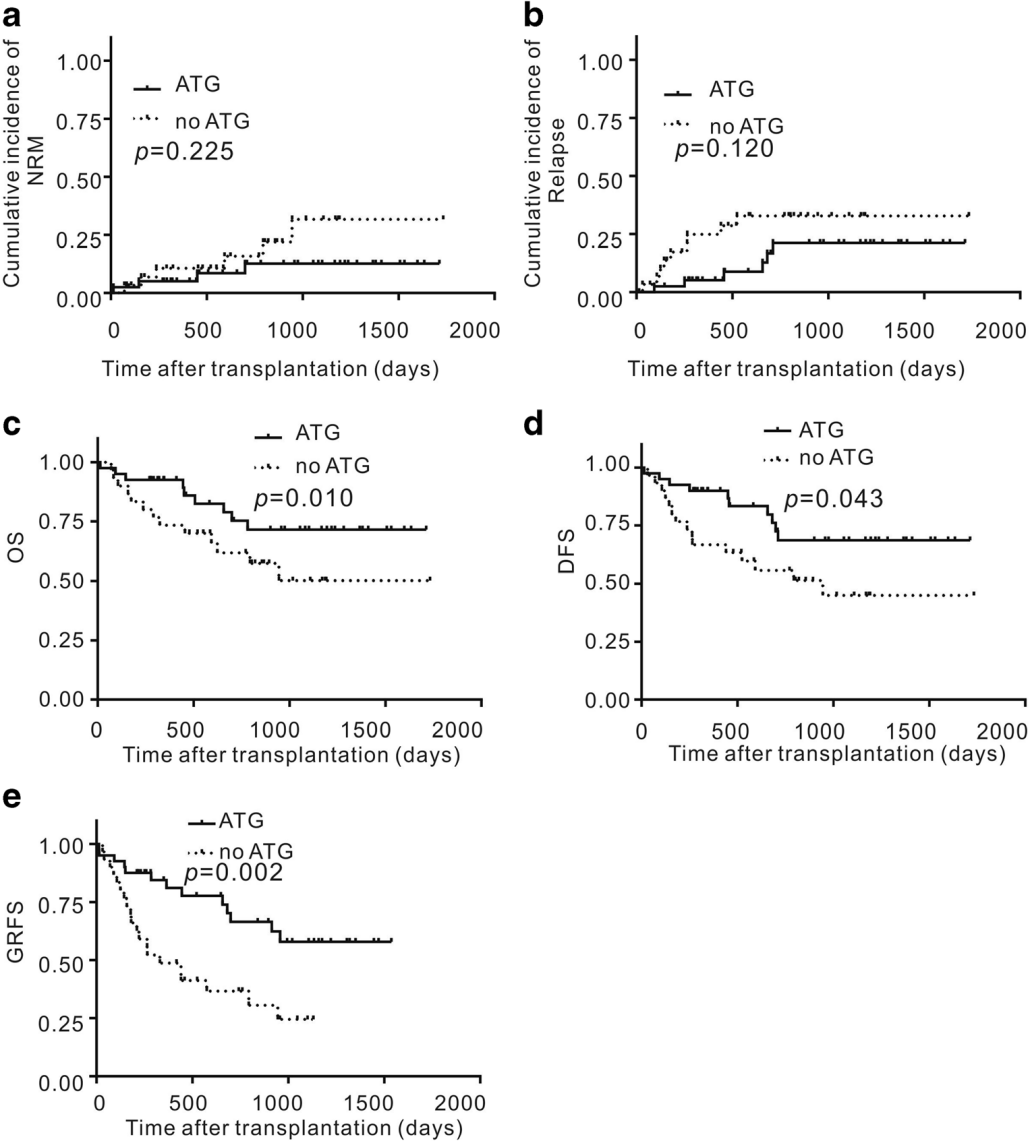
Cumulative incidence of TRM, relapse, OS, DFS, and GRFS after MSD-PBSCT with low-dose rATG or no rATG in combination with cyclosporine, mycophenolate, and short-term methotrexate for GVHD prophylaxis. NRM, means treatment-related mortality; OS, overall survival; DFS, disease-free survival; GRFS, GVHD-free relapse-free survival; GVHD, graft-versus-host disease

In the ATG group, non-relapse mortality occurred in 4 (10.0%) of the 40 patients who received allogeneic MSD-PBSCT. Among these, no patients died of GVHD, 3 patients died of pneumonia and respiratory failure, and 1 patient died of gastrointestinal hemorrhage before engraftment. The 6-month, 1-year, and 2-year cumulative incidence of TRM in the ATG group was 5.8% (95% CI, 1.1–23.7%), 9.5% (95% CI, 2.5–38.0%), and 14.0% (95% CI, 13.5–39.9%), respectively (Fig. 1c). The 2-year cumulative incidence of TRM in the ATG and no-ATG groups was not significantly different (14.0% (95% CI, 13.5–39.9%) in ATG group vs. 31.6% (95% CI, 3.6–51.7%) in no-ATG group; *P* = 0.225) (Fig. 3a).

In the ATG group, 5 patients (12.5%) who received allogeneic MSD-PBSCT relapsed at a median of 428 (93–682) days after transplantation. The 6-month, 1-year, and 2-year cumulative incidence of relapse was 6.8% (95% CI, 2.8–24.6%), 18.1% (95% CI, 5.5–34.0%), and 22.6% (95% CI, 20.1–38.9%), respectively (Fig. 1d). Of the five patients with relapse after transplantation, three patients were diagnosed as MDS EB-2 before transplantation, and two patients were diagnosed as T lymphoblastic lymphoma (CR1) and MLL-AF9 AML (CR1). All the relapsed patients died at a median of 87 (31–195) days after relapse, even though they were treated with salvage therapy. On univariate analysis, high-risk disease, according to the Disease Risk Index, was a risk factor for relapse (Table 3). The 2-year cumulative incidence of relapse in the ATG and no-ATG groups was not significantly different (22.6% (95% CI, 20.1–38.9%) in the ATG group vs. 32.8% (95% CI, 12.5–48.4%) in the no-ATG group; *P* = 0.120; Fig. 3b).

### Survival and the quality of life

In the ATG group, median follow-up after transplantation among survivors was 861 (271–1622) days. At the time of analysis, 31 (77.5%) patients were still alive in CR. Kaplan–Meier estimates of OS 1 year, 2 years, and 3 years after transplantation were 93.1% (95% CI, 75.1–98.2%),73.1% (95% CI, 53.1–85.7%), and 68.9% (95% CI, 48.2–82.7%; Fig. 1e); DFS 1 year, 2 years, and 3 years after transplantation were 91.4% (95% CI, 75.5–97.1%), 68.9% (95% CI, 48.2–82.7%), and 68.9% (95% CI, 48.2–82.7%; Fig. 1F); and GRFS 1 year, 2 years, and 3 years after transplantation were 78.9% (95% CI, 60.5–89.4%), 64.4% (95% CI, 44.5–78.7%), and 54.0% (95% CI, 33.0–71.0%), respectively.

The 3-year OS rate was 68.9% (95% CI, 48.2–82.7%) for patients in the ATG group and 50.2% (95% CI, 33.0–76.2%) for patients in the no-ATG group (*P* = 0·010; Fig. 3c). The 3-year DFS rate was 68.9% (95% CI, 48.2–82.7%) for patients in the ATG group and 44.9% (95% CI, 28.7–70.3%) for patients in the no-ATG group (*P* = 0.043; Fig. 3d). The 3-year GRFS rate was 54.0% (95% CI, 33.0–71.0%) for patients in the ATG group and 24.4% (95% CI, 11.5–51.8%) for patients in the no-ATG group (*P* = 0·002; Fig. 3e). On univariate and multivariate analyses, no-ATG was a risk factor for DFS and GRFS (*P* < 0.05) (Tables 4 and 5). On univariate and multivariate analyses, high-risk disease, according to the Disease Risk Index, was a risk factor for poor OS or DFS in the ATG group (Table 2).

**Table 5:** Univariate and multivariate analyses of OS, GRFS, DFS, or GVHD for the risk factors of transplant outcomes in all patients

	Univariate			Multivariate		
	HR	95% CI	*P*	HR	95% CI	*P*
GRFS						
Group						
No ATG	1.00			1.00		
ATG	0.22	0.20–0.70	0.00	0.30	0.14–0.65	0.00
OS						
Group						
No ATG	1.00			1.00		
ATG	0.50	0.20–1.20	0.10	0.16	0.03–0.76	0.02
DFS						
Group						
No ATG	1.00			1.00		
ATG	0.40	0.20–0.89	0.04	0.04	0.00–0.35	0.00
Chronic GVHD						
Group						
No ATG	1.00			–		
ATG	0.37	0.05–2.58	0.31	–	–	–
Extensive chronic GVHD						
Group						
No ATG	1.00			1.00		
ATG	0.30	0.10–0.80	0.02	0.02	0.00–0.26	0.00

*ATG*, anti-thymocyte globulin; *95% CI*, cumulative incidence 95% CI; *DFS*, disease-free survival; *GVHD*, graft-versus-host disease; *GRFS*, severe GVHD-free, relapse-free survival; *OS*, overall survival

Chronic, limited, and extensive grades of GVHD were defined according to the Seattle criteria

Chronic, mild, moderate, and severe grades of GVHD were defined according the NIH criteria

^*^P value

In the ATG group, health of survivors, assessed with the Karnofsky Performance Scale Index, showed 26 patients had scores of 90–100, 4 patients had a score of 80 due to cGVHD, and 1 patient had a score of 20 due to cerebral hemorrhage.

### T cell immune reconstitution

**Fig. 4:**
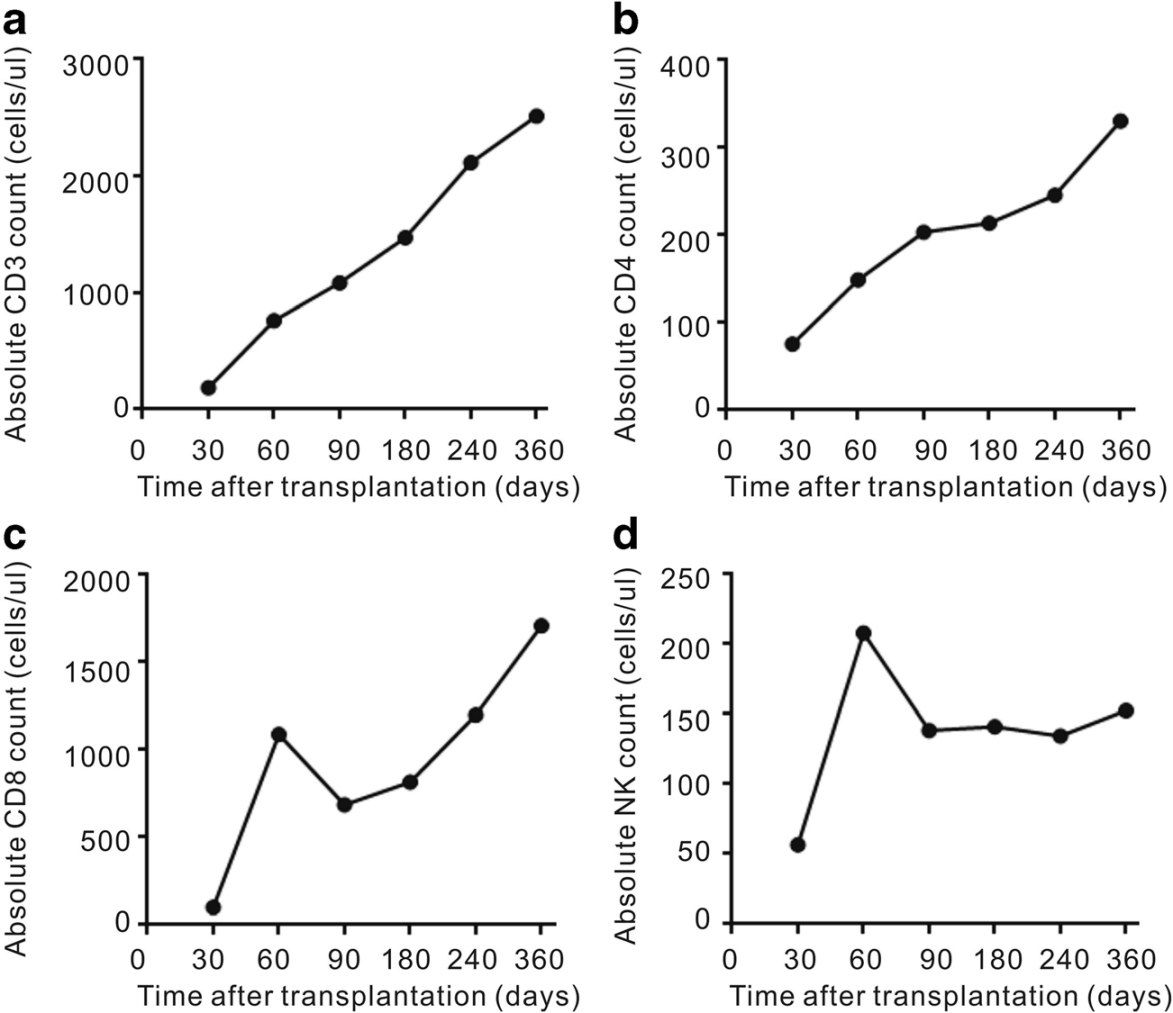
Lymphocyte counts, stratified into CD3^+^, CD4^+^, CD8^+^, and CD56/CD16^+^ subpopulations, at days +30, +60, +90, +180, +240, and +360 after MSD-PBSCT with low-dose rATG in combination with cyclosporine, mycophenolate, and short-term methotrexate for GVHD prophylaxis

In the ATG group, median lymphocyte counts, stratified into CD3^+^, CD4^+^, CD8^+^, and CD56/CD16^+^ subpopulations, are depicted in Fig. 4. Seventeen patients were included in immune reconstitution studies, and 14 patients had evaluable data at all time points. On day +100, median CD3^+^, CD4^+^, CD8^+^, and CD56/CD16^+^ counts were 914 (642–1465), 189 (63–488), 686 (483–1355), and 138 (75–250), respectively. CD4^+^ cell counts were > 200/μL at day +120 and reached 330/μL on day +365. There was no association between CD3^+^, CD4^+^, CD8^+^, and CD56^+^ cell counts at 1, 2, 3, 6, and 12 months and relapse, the occurrence of GVHD, CMV/EBV reactivation, or TRM.

On day +100 in the no-ATG and ATG groups, median CD3^+^, CD4^+^, CD8^+^, and CD56/CD16^+^ counts were 1600 (768–2137) and 914 (642–1465), 210 (201–274) and 189 (63–488), 878 (290–1490) and 686 (483–1355), and 315 (111–546) and 238 (75–350)/μL, respectively. There were no differences between the two groups.

## Discussion

cGVHD is the leading cause of non-relapse morbidity and mortality after allogeneic PBSCT [19]. Strategies aimed at decreasing the impact of moderate to severe cGVHD have limited efficacy, and prophylaxis is considered a superior option. This study investigated the feasibility of a 5 mg/kg total dose of rATG administered over 2 days (days −5 to −4) for cGVHD prophylaxis in patients receiving allogeneic MSD-PBSCT. Findings showed the incidence of extensive cGVHD and severe cGVHD was low. The health of the survivors, assessed by a physician using the Karnofsky Performance Scale Index, was good, and immunosuppressive drugs were withdrawn in 93.5% of patients within 3 years after transplantation.

Optimizing the dose and timing of ATG before and after hemopoietic cell transplantation is essential to enhance patient outcomes. Kroger et al. investigated the use of ATG (rATG-F; ATG-Fresenius) at a dose of 10 mg/kg on 3, 2, and 1 days before transplantation of allogeneic peripheral blood stem cells from an HLA-identical donor in patients with acute leukemia [2]. The rate of grades 2–4 aGVHD was 10.8%, the 2-year cumulative incidence of cGVHD was 32.2%, and the 2-year cumulative incidence of clinical extensive cGVHD was 7.6% [2]. They used a highly purified rabbit polyclonal antihuman T lymphocyte immunoglobulin resulting from immunization of rabbits with the Jurkat T-lymphoblast cell line. Rubio et al. reported that the use of ATG followed by HLA-identical sibling donor allogeneic stem cell transplantation reduced the incidence of cGVHD without increasing the risk of relapse; however, this study was limited as dose, duration, brand of ATG, and GVHD prophylaxis regimen were not reported [7]. Other ATGs derived from rabbits or horses have been used in unrelated matched stem cell transplantation, but the different brands and doses are not interchangeable because of their various immunologic properties [7–11]. High exposure of ATG before transplantation resulted in significantly reduced incidence of graft failure and extensive cGVHD [20]. However, compared with exposure of ATG before transplantation, exposure of ATG after allogeneic stem cell transplantation was less effective for the prevention of grade 2–4 aGVHD and extensive cGVHD, but was essential for early immune reconstitution [21]. Socie et al. [3] found that risk of relapse did not increase with thymoglobulin doses < 6 mg/kg. In the present study, rATG was received at an intravenous dose of 5 mg/kg divided over 2 days starting early on day −5 to increase exposure of rATG before transplantation and ensure low exposure of rATG after transplantation. The 2-year cumulative incidences of grade 2–4 aGVHD, grade 3–4 aGVHD, extensive cGVHD, severe cGVHD, and TRM were 30.0%, 2.6%, 11.4%, 14.7%, and 14.0%, respectively. In patients who received MSD-PBSCT in our unit prior to January, 2013, the incidence of grade 2–4 aGVHD, grade 3–4 aGVHD, extensive cGVHD, severe cGVHD, and TRM was 13.9%, 9.8%, 41.5%, 21.2%, and 10.2%, respectively. Although the incidence of GVHD between these two cohorts cannot be directly compared, the data imply that rATG 5 mg/kg administered on days −5 to −4 has potential to reduce the incidence of severe or extensive cGVHD without increasing the risk of relapse.

rATG contains polyclonal immunoglobulins obtained from hyper-immune sera of rabbits immunized with human thymocytes [22, 23]. rATG administered before stem cell infusion can persist for long periods in vivo and cause T cell depletion of the donor graft [24], but also host immunosuppression, infection, and relapse [25]. Successful immune reconstitution after transplantation is critical for minimizing relapse and TRM [26, 27]. In a large study investigating the relationship between ATG exposure, CD4^+^ T cell immune reconstitution, and clinical outcomes following pediatric cord blood transplantation (CBT), early CD4^+^ immune reconstitution after CBT improved event-free survival. Early CD4^+^ immune reconstitution was achieved by reducing exposure to ATG after CBT [20]. CD4^+^ T cells > 200/μL at 3 months post-transplantation have been associated with superior OS and TRM. In the present study, we observed rapid recovery of CD3^+^ T cell and CD4^+^ T cells and a low rate of infectious complications and relapse. On day +100, median CD3^+^ and CD4^+^ T cell counts were 1117 and 104/μL. CD4^+^ cell counts were > 200/μL at day +120 and reached 330/μL on day +365.

In summary, these data suggest that rATG for GVHD prophylaxis is tolerable and efficacious at a 5 mg/kg total dose administered over 2 days (day −5 to −4) in patients receiving allogeneic MSD-PBSCT. Further studies are required to evaluate the active rATG serum concentrations and the optimal prophylactic strategy in this patient population.
